# Therapeutic potential of peptidomimetics of suppressors of cytokine signaling (SOCS) proteins in rheumatic disorders

**DOI:** 10.3389/fmed.2025.1708293

**Published:** 2025-11-28

**Authors:** Alessia Cugudda, Daniela Marasco

**Affiliations:** Department of Pharmacy, University of Naples Federico II, Naples, Italy

**Keywords:** inflammation, SOCS, rheumatic disorders, peptidomimetics, KIR, cytokine, JAK/STAT pathway

## Abstract

Dysregulation of the Janus Kinase (JAK)/Signal Transducer and Activator of Transcription (STAT) pathway is increasingly recognized as a central molecular hallmark in the pathogenesis of multiple rheumatic diseases. Suppressors of cytokine signaling (SOCS) proteins function as critical intracellular inhibitors of JAK/STAT signaling through a classical negative feedback mechanism. In Rheumatoid Arthritis (RA), aberrant upregulation of SOCS1 and SOCS3 has been documented in peripheral blood T lymphocytes, monocytes, and synovial tissues, with expression levels correlating with disease activity and progression. Notably, diminished basal expression of SOCS1 mRNA is associated with poor therapeutic response to methotrexate or rituximab, and specific SOCS1 polymorphisms have been genetically linked to RA susceptibility. In Ankylosing Spondylitis (AS), enhanced SOCS3 expression in Peripheral Blood Mononuclear Cells (PBMCs), CD4^+^ T cells, and monocytes show positive correlation with systemic inflammatory markers such as Erythrocyte Sedimentation Rate (ESR) and C-Reactive Protein (CRP), as well as with clinical indices of functional impairment. Conversely, SOCS1 expression is attenuated in T cells during phases of low-grade inflammation, suggesting context-dependent regulatory dynamics. In drug discovery for inflammatory diseases, recent advances have focused on the development of SOCS peptidomimetics, particularly those derived from the Kinase Inhibitory Region (KIR) of SOCS1, as novel immunomodulatory agents. These compounds have been shown to modulate hyperactive JAK/STAT signaling in autoimmune conditions. In this perspective article, we analyze current progress in the development and preclinical evaluation of mimetics of SOCS proteins and discuss their prospective role in the treatment paradigm for rheumatic disorders. Herein, we propose that peptidomimetics of SOCSs may represent a new frontier in the precise modulation of JAK/STAT signaling, offering a promising avenue toward personalized prevention and treatment of rheumatic pathologies.

## 1 Introduction

**FIGURE 1:**
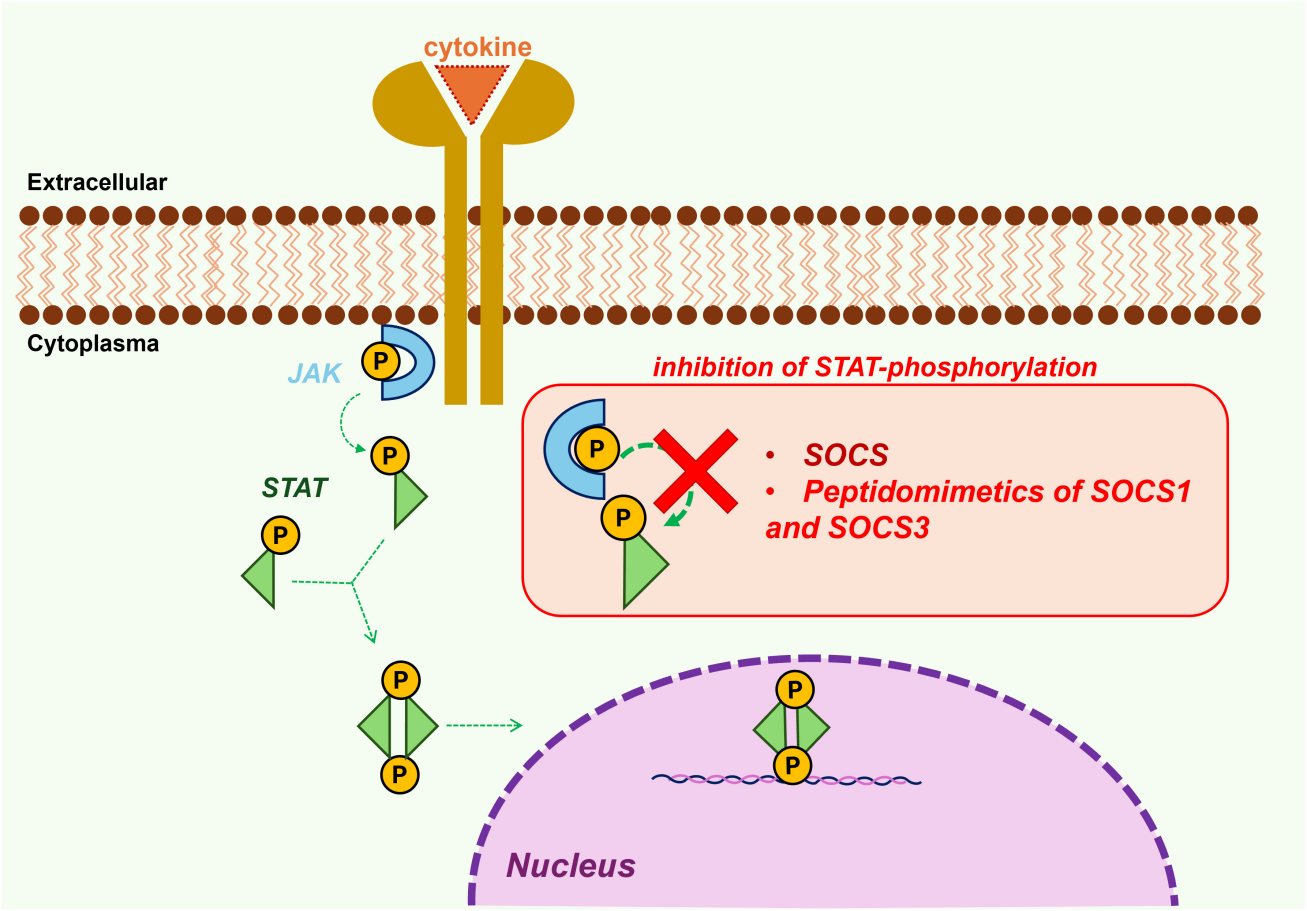
Schematic representation of cytokine-induced activation of the Janus Kinase (JAK)/Signal Transducer and Activator of Transcription (STAT) signaling pathway and its negative regulation by suppressors of cytokine signaling (SOCS) proteins and SOCS peptidomimetics, highlighting their potential to modulate inflammatory responses.

Suppressors of cytokine signaling proteins are crucial regulators of the JAK/STAT pathway, whose aberrant activation drives exaggerated immune and inflammatory responses, which are hallmarks of rheumatic diseases (1). Furthermore, patients diagnosed with rheumatic diseases, including RA, SLE, and AS, demonstrate an elevated probability of developing comorbid conditions such as diabetes (2–4), atherosclerosis (5), and ocular inflammatory diseases (6, 7). This increased risk is primarily attributable to chronic systemic inflammation and the utilization of corticosteroids or JAK inhibitors (JAKi). Concurrently, these conditions exhibit dysregulation of the JAK/STAT signaling pathway, a factor that may contribute to their pathophysiology. Consequently, a promising therapeutic strategy is to restore basal SOCS protein levels exogenously, thereby harnessing their role as negative feedback regulators of this pathway (8). The Mechanism Of Action (MOA) of the SOCS proteins is characterized by the inhibition of the JAK/STAT pathway, primarily through competitive binding of their SH2 domains with those of Signal Transducers and Activators of Transcription (STATs), thereby preventing their interaction with JAK kinases (Figure 1) (9). Beyond this general mechanism, the SOCS1 and SOCS3 proteins, which are the family members most closely linked to rheumatic diseases, harbor a Kinase Inhibitory Region (KIR) that functions as a pseudo-substrate of the catalytic site of JAKs, directly suppressing kinase activity (10). In addition, they bind JAKs, in different forms since SOCS1 forms a binary complex, while SOCS3 forms a ternary complex involving Glycoprotein 130 (Gp130) (11).

## 2 Role of SOCS in rheumatoid arthritis (RA)

Rheumatoid arthritis has been identified as a global epidemic, as evidenced by a recent study which reported an increase in the global incidence rate from 11.66 to 13.48 per 100,000 population (12). RA shows higher incidence in women, with smoking as a major risk factor. The disease burden is greater in regions with limited healthcare due to poor prevention and delayed diagnosis (12). RA is a chronic autoimmune disease driven by dysregulated immune responses, where activated lymphocytes, pro-inflammatory cytokines and autoantibodies [e.g., anti-Cyclic Citrullinated Peptide (CCP)] promote tissue damage (13). The course of RA is characterized primarily by synovial inflammation, leading to symptoms including pain, stiffness, and joint swelling with a predilection for the hands, wrists and feet (14, 15). Given the incurable nature of the condition, novel therapeutic interventions are being developed with the objective of reducing inflammation and enhancing patients’ quality of life (16). The analysis of SOCS proteins expression in RA offers valuable insights into mechanisms of inflammation control and pain reduction, given their altered expression patterns, which vary by cell type and subcellular localization (17). SOCS1 is a major regulator in RA and contributes to maintaining immune homeostasis, a process frequently compromised in RA (18). Its expression is significantly upregulated in peripheral T cells and PBMCs, suggesting a potential role in limiting excessive inflammatory responses (17, 19). Activation of SOCS1 suppressed key pro-inflammatory cytokines, including interleukin-6 (IL-6), interleukin-17 (IL-17), and tumor necrosis factor-alpha (TNF-α), thereby limiting T helper 17 (Th17) cell activation and reducing joint inflammation. This effect was observed following treatment with shikonin, a naphthoquinone compound known to inhibit STAT1/STAT3 signaling by upregulating the expression of SOCS1 (20). These findings support the potential of SOCS1-based therapeutic strategies as novel interventions for RA (21). Conversely, SOCS3 is overexpressed in macrophages and synovial fibroblasts in patients with RA, where it appears to contribute to Th17 cell resistance, exacerbating tissue damage. However, this overexpression is hypothesized to be a compensatory mechanism due to excessive IL-6-mediated STAT3 activation, which prevents SOCS3 from performing its usual anti-inflammatory role (17). Experimental studies on macrophage models and joint fibroblasts have demonstrated that SOCS3, by effectively inhibiting STAT3 activation, can attenuate the severity of RA (22). Furthermore, given that these alterations in SOCS3 expression are insufficient to control the excess inflammatory signal and activate the compensatory mechanism, one option would be to enhance SOCS3 via the cyclic Adenosine Monophosphate (cAMP)/Exchange protein directly activated by cAMP (Epac)/Ras-related protein 1 (Rap1)/SOCS3 cAMP pathway. This could therefore represent a strategy for limiting inflammatory damage (23). The enhancement of the expression of SOCS1 or SOCS3 has shown therapeutic potential; however, caution is warranted given the context-dependent and potentially dual role of SOCS3 (21, 24). Several JAK inhibitors are being evaluated in ongoing clinical trials; in particular upadacitinib is being assessed for efficacy and safety in patients with RA who are refractory to biologic Disease-Modifying Antirheumatic Drugs (bDMARDs), where it has been consistently demonstrated to be effective (25, 26).

## 3 Role of SOCS in Ankylosing Spondylitis (AS)

Ankylosing Spondylitis is a chronic inflammatory disorder within the spondylarthritis spectrum that predominantly affects the axial skeleton, leading to structural deformities and progressive functional impairment (27). The disease arises from an aberrant immune response in which the joints and spine are misidentified as non-self, resulting in chronic inflammation, severe pain, and reduced mobility (28). From an epidemiological perspective, AS manifests with a higher prevalence in males, typically manifesting between the ages of 20 and 40, and has an overall prevalence ranging from 0.1% to 1.4% within the global population (29). With non-curative options available, efforts are focused on targeted therapies to overcome the limitations of Non-Steroidal Anti-Inflammatory Drugs (NSAIDs)-based management. Emerging evidence suggests that the IL-23/IL-17/STAT3 signaling pathway is crucial in the development of AS (30). JAK/STAT pathway sustains chronic inflammation by activating Th17 cells, with STAT3 acting as a key driver by promoting pro-inflammatory gene expression and Th17 survival (31). Dysregulated STAT3 activity can lead to excessive tissue inflammation and damage, linking AS to a broader spectrum of immune-mediated disorders. Particularly, in experimental models of SpondyloArthritis (SpA) (a family of chronic inflammatory diseases with AS as a major subtype), treatment with a Protein Inhibitor of Activated STAT3 (PIAS3) significantly improved histological outcomes, suppressed IL-17, TNF-α, and STAT3 expression, and promoted a T cell shift favoring regulatory T cells (Treg) over Th17 cells (30). In this context, SOCS3 has attracted attention for its inhibitory action on the STAT3 pathway. Recent findings have shown that SOCS3 promotes Th17 differentiation and contributes to abnormal bone formation, with its overexpression consistently observed in PBMCs, CD4^+^ T cells, and monocytes of AS patients, where SOCS1 appears to be downregulated and its exogenous administration may help limit T cell differentiation and inflammation (32–34). Rather than worsening the disease, the upregulation of SOCS3 may reflect an intrinsic compensatory mechanism to counteract chronic hyperinflammation. This paradox underscores the dual, context-dependent role of SOCS3 in AS pathogenesis and suggests that targeting the STAT3/SOCS3 axis might help restore immune balance.

## 4 Role of SOCS in systemic lupus erythematosus (SLE)

Systemic lupus erythematosus is a chronic autoimmune disorder with a multifactorial etiology that integrates genetic susceptibility, immune dysregulation, hormonal influences, and environmental triggers (35). The global incidence of SLE is estimated at 5.14 cases per 100,000 person-years, corresponding to approximately 0.4 million newly diagnosed individuals annually (36). The disease predominantly affects women of childbearing age, accounting for nearly 90% of cases (37). Its immunopathogenesis is marked by aberrant activation of both innate and adaptive immune pathways. T-cell hyperactivation drives abnormal B-cell responses, resulting in excessive autoantibody production and the formation of immune complexes which deposit in tissues and initiate a cascade of inflammatory events. Target organs include the skin, joints, kidneys, heart, lungs, and central nervous system (35). Although no curative therapies exist, current treatments focus on symptom relief, prevention of organ damage, and improved long-term survival. Hydroxychloroquine continues to be the most significant drug in this field, due to its ability to influence the immune system by increasing lysosomal pH, inhibiting TLR7/9 signaling, reducing IFN-I production and pro-inflammatory cytokine responses (IL-6) (38). This, in turn, limits the activation of autoreactive immune cells (37). SOCS proteins have gained increasing attention in SLE, with SOCS1 emerging as the most consistently involved. Its downregulation is frequently observed and contributes to the dysregulated interferon (IFN) pathway and the characteristic “interferon signature” of the disease. Experimental and clinical evidence links reduced SOCS1 activity to the development of autoimmune conditions such as RA, SLE, vasculitis, and Sjögren’s syndrome, underscoring its key regulatory role (39–41). Given its function in modulating the IFN/JAK/STAT axis, SOCS1 represents a promising therapeutic target. Preclinical studies on KIR-SOCS1 peptidomimetics have demonstrated efficacy in suppressing lymphocyte activation and reducing lupus pathology, potentially complementing current JAK inhibitor strategies (42–44). Several JAK inhibitors (baricitinib, tofacitinib, upadacitinib, and deucravacitinib) are currently under clinical investigation in SLE (45–47). Simultaneously, SOCS3 expression in podocytes has been shown to exert a protective effect in glomerulonephritis development and to inhibit autoantibody production in the imiquimod-induced lupus model, presumably through suppression of IL-6 production in podocytes (48). However, its role appears to be context-dependent, as both protective and pathogenic effects have been reported (49). These findings suggest that, as in other autoimmune disorders, the peptidomimetics of SOCS proteins may represent promising therapeutic targets (50).

## 5 Discussion

The potential of peptides as therapeutic agents in inflammatory and autoimmune diseases (51), including rheumatic conditions, stands out critically when examined against established rivals such as JAKi and small molecules. While JAKi provide important practical benefits, in particular rapid oral bioavailability and efficient intracellular targeting, they carry considerable safety concerns, including increased cardiovascular, and thromboembolic risks and oncogenic potential due to disruption of immune surveillance via the JAK/STAT pathway (Table 1) (52, 53).

**TABLE 1:** Schematic comparison: direct JAK inhibitors (JAKi) vs. suppressors of cytokine signaling (SOCS) peptidomimetics.

Parameters	JAKi	SOCS peptidomimetics
MOA	Bind ATP-binding site of JAKs → block kinase activity globally	Mimic SOCS KIR and SH2 domains → pseudo-substrate inhibition of JAK catalytic site + competitive blockade of STAT docking
MOI	Direct, broad, and continuous blockade of all downstream STAT signaling	Feedback-mimetic, context-dependent modulation — acts preferentially in hyperactivated JAK/STAT environments
Specificity	Limited — affects multiple JAK isoforms and cytokine pathways (IL-6, IFN, GM-CSF, etc.)	High — targets disease-relevant JAK/STAT nodes (e.g., STAT1 in RA/SLE; STAT3 in AS) while preserving basal signaling
Intracellular regulation	External pharmacological block, independent of physiological control	Mimics endogenous negative-feedback loop; restores physiological regulation
Immune modulation	Global cytokine suppression → broad immunosuppression	Selective rebalancing of Th17/Treg axis, macrophage polarization, and cytokine tone
Safety profile	Risks: infections, thrombosis, malignancy due to loss of immune surveillance	Predicted lower systemic toxicity; avoids chronic immune suppression by acting locally and transiently
Therapeutic concept	“Turn off” inflammation	“Recalibrate” inflammation via physiologic feedback
Drug type	Small molecule (ATP-site inhibitor)	Peptide or peptidomimetic
Delivery	Oral; systemic	Intracellular, often nanoparticle-encapsulated or cell-penetrating peptide–based
Representative drugs	Baricitinib, Tofacitinib, Upadacitinib	SOCS1-KIR, R9-SOCS1-KIR, PS5, icPS5, SOCS3-KIRESS

In general, small molecules have the advantages of oral delivery and cost-effective production but struggle with targeting extended Protein–Protein Interactions (PPIs), resulting in off-target effects and reduced selectivity (54) and can be associated with notable safety concerns, specifically the potential to increase cardiovascular risk. The JAKi effectively block the ATP-binding site of one or more JAK isoforms, but this mechanism inevitably results in broad intracellular kinase inhibition, suppressing multiple cytokine signals simultaneously. In this context, both the EMA and FDA have issued warnings recommending caution in their use (52). In sharp contrast, peptide-based therapies achieve higher specificity and affinity, especially in modulating PPIs (55). Their natural amino acid composition enhances safety by avoiding tissue accumulation (56) while design flexibility allows chemical modifications (e.g., D-amino acids, PEGylation) to optimize stability and solubility (57). Emerging delivery techniques, such as nanoparticle encapsulation and Cell-Penetrating Peptides (CPP) (58, 59), promise to overcome historical peptide delivery challenges, improving targeting and minimizing side effects (60). The employment of SOCS peptidomimetics represents an immunoregulatory approach that aligns with endogenous feedback biology. By mimicking the selective, reversible inhibition of JAK activity of natural SOCS proteins, these compounds offer a potential therapeutic approach to limit inflammation without resulting in global immunosuppression (61). Their ability to modulate STAT signaling in a context-dependent manner (i.e., mainly when the pathway is hyperactivated) has the potential to address the tissue-specific pathology characteristic of rheumatic diseases, including synovial hyperplasia, cartilage degradation and aberrant bone formation, with greater safety and efficacy than current biologics or JAKi. These agents bind to JAKs (KIR pseudo-substrates) and/or compete at STAT-recruitment surfaces (SH2-like interactions), thereby more closely reproducing physiological inhibition (62). SOCS3-enhancing peptides and SOCS3 gene-transfer strategies demonstrated disease-modifying activity in murine Collagen-Induced Arthritis (CIA) models, including reduced joint destruction and attenuation of inflammatory responses (63, 64). For SOCS1 mimetics, robust evidence is available from autoimmune disease models such as lupus, and mechanistic studies in arthritis models (e.g., SOCS1-deficient mice) consistently support their protective role (24, 39, 65). However, studies directly assessing the therapeutic administration of peptidomimetics of SOCS1 in fully developed arthritis models remain limited. In their mechanisms of inhibition (MOIs), JAKi are widely used to treat arthritis because they block JAK/STAT signaling and reduce the downstream production of inflammatory cytokines and limit the activation of synovial fibroblasts, which are key effectors of persistent joint inflammation (66) (Table 1). However, suppressing IL-6/STAT3 and IFN-dependent cascades in the synovium, although anti-inflammatory, also compromises reparative signaling (67). In contrast, peptidomimetics of SOCS1 and SOCS3 restore physiological negative feedback by reducing IL-6, IL-17, and TNF-α but maintaining tissue homeostasis.

In cartilage, JAKi blunt cytokine-driven catabolism but also disrupt JAK-dependent anabolic pathways (67, 68), in contrast, SOCS peptidomimetics are more selective since they inhibit STAT1/3 activity, restore redox balance (69), and preserve matrix integrity in arthritic chondrocytes via SOCS3 modulation (70) (Table 1). At the enthesis, JAKi only partially suppress IL-23/IL-17-mediated inflammation and fail to counteract pathological osteogenesis, whereas SOCS3 modulation limits STAT3-driven bone formation (71, 72). Immune cells further highlight these divergent mechanisms: JAKi broadly suppress Th1/Th17/Treg responses, which contributes to their clinical efficacy but also increases susceptibility to infection. In contrast, SOCS1 reduces STAT1/3 activity and rebalances Th17/Treg differentiation, whereas SOCS3 mimetics modulate macrophage and T-cell polarization with far greater precision and without inducing broad immunosuppression (73–75). Instead, the therapeutic effectiveness of JAKi is increasingly tempered by their association with cardiovascular and oncogenic risks (76–78). Peptidomimetics of SOCS, by mimicking endogenous checkpoint control, achieve context-dependent and tissue-specific inhibition with a more favorable safety profile (79).

Thus, from a forward-looking perspective, novel regulatory peptides inspired by SOCSs provide a compelling route to innovate rheumatic disease treatments.

**FIGURE 2:**
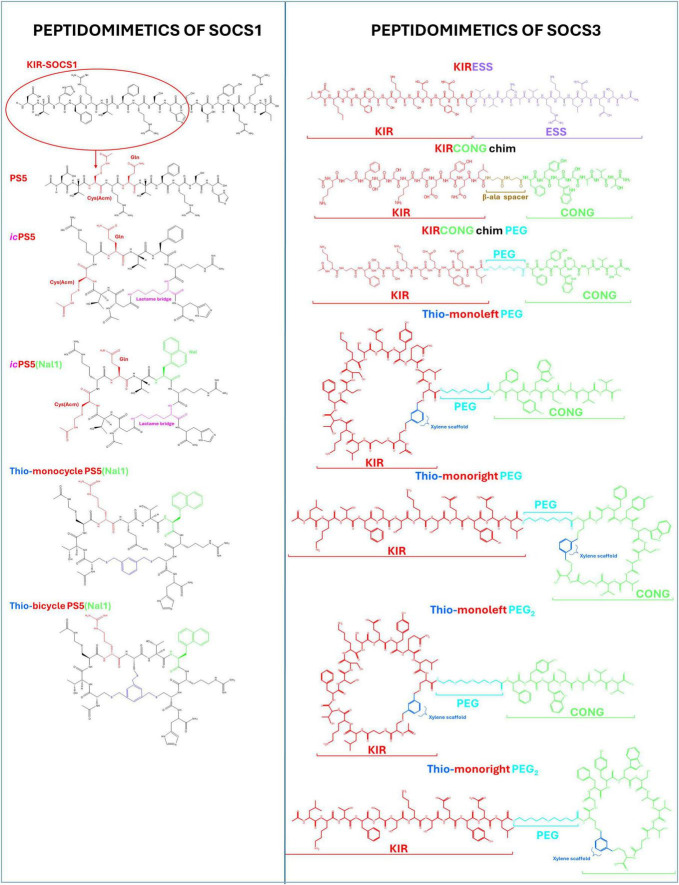
Schematic structures of suppressors of cytokine signaling (SOCS) peptidomimetics: left panel for SOCS1, Cys(Acm) and Gln residues are highlighted in red, Nal in green, the lactam bridge in fuchsia, and the xylene scaffolds in blue; right panel for SOCS3, Kinase Inhibitory Region (KIR) sequence is in red, the ESS domain is in violet, the CONG domain is in green, xylene scaffolds are in blue, the β-alanine spacer in brown and the PEG moieties in azure.

Peptidomimetics based on SOCS1 and SOCS3 have demonstrated selective inhibition of JAK catalytic activity, suppression of pro-inflammatory signaling pathways and the progressive development of peptidomimetics of SOCS1 and SOCS3 illustrates a paradigm shift in how intracellular checkpoints of cytokine signaling can be pharmacologically harnessed (80). Early studies with the SOCS1-KIR peptide (Figure 2) established a foundational concept: selective interference with JAK2 activity could effectively silence STAT1-driven inflammation (81). When equipped with an arginine-rich CPP sequence (R9-SOCS1-KIR), the peptide demonstrated not only cell permeability but broad immunomodulatory and antioxidant capacity *in vivo*. Its ability to attenuate ocular and systemic inflammatory responses in diverse models -from autoimmune uveitis to diabetic nephropathy- highlighted a mechanism that extends beyond cytokine inhibition to the restoration of redox balance and tissue homeostasis (82–84).

The subsequent optimization of KIR into the PS5 peptidomimetic (Figure 2) reflected a rational effort to stabilize and potentiate this interaction. By condensing the KIR motif into a compact 10–amino acid sequence with enhanced JAK2 affinity, PS5 offered both mechanistic clarity and therapeutic promise. Its dual suppression of STAT-dependent transcription and oxidative gene expression positioned it as a prototype for targeted anti-inflammatory design (62, 85). The transition from PS5 to cyclic derivatives such as icPS5 and *ic*PS5(Nal1) (Figure 2) further demonstrated how structural constraints can amplify potency, stability, and biological persistence. The observation that aromatic modifications can stabilize binding to JAK2 underscores the intricate role of peptide chemistry in achieving both specificity and durability of action (86–88). These advances collectively reveal that SOCS1 mimetics act not merely as inhibitors but as molecular modulators that recalibrate dysregulated inflammatory and oxidative networks. Their effects in metabolic, vascular, and autoimmune contexts suggest that mimicking intrinsic feedback regulators can achieve therapeutic modulation with reduced off-target burden compared to conventional kinase inhibitors. Parallel efforts in SOCS3 mimicry have expanded this framework. The design of KIRESS and chimeric KIRCONG peptides, integrating KIR, ESS, and CONG regions (Figure 2), extends the strategy for the development of SOCS3 mimetics toward STAT3-driven pathologies (89, 90).

Many challenges are still inherent to mimetic therapeutics in particular, SOCS3 analogues demonstrate how minor alterations in topology or hydrophobicity can influence both target engagement and formulation viability. While CLIPS-based xylene scaffolds represent a step toward resolving the issues of structural rigidity and partial flexibility, the persistent solubility issues point to the need for hybrid chemistries that integrate peptide and small-molecule features (91, 92).

However, strident challenges remain: (i) optimal aqueous solubility and bioavailability of peptidomimetics, (ii) the balance between conformational rigidity for affinity and flexibility for binding site adaptation. Moreover, robust preclinical and clinical validation of SOCS mimetics as therapeutics is essential to establish safety, efficacy, and comparative advantages over established small molecules and JAKi. In summary, the future therapeutic landscape anticipates peptides not merely as alternatives but as transformative agents uniquely positioned to overcome the limitations of JAKi and small molecules. Their ability to precisely modulate complex PPIs implicated in inflammation, together with advances in chemical design and delivery, herald a new paradigm in targeting autoimmune and rheumatic diseases resistant to current treatments. This critical analysis highlights the potency of peptides shaped by structural insights from endogenous regulators, in this case SOCS proteins, as a frontier in rheumatology drug development, emphasizing the need for optimized formulations and comprehensive translational studies toward clinical application.

## Data Availability

The original contributions presented in this study are included in this article/supplementary material, further inquiries can be directed to the corresponding author.
